# Predicting *Phloeosinus cupressi* (Coleoptera: Curculionidae: Phloeosinus) Distribution for Management Planning Under Climate Change

**DOI:** 10.3390/insects17010077

**Published:** 2026-01-09

**Authors:** Yu Cao, Kaitong Xiao, Lei Ling, Qiang Wu, Beibei Huang, Xiaosu Deng, Yingxuan Cao, Hang Ning, Hui Chen

**Affiliations:** 1 Hubei Key Laboratory of Biological Resources Protection and Utilization, Hubei Minzu University, Enshi 445000, China; zhedaotinihui@163.com (Y.C.); lxwq20030226@163.com (Q.W.); hbei0825@163.com (B.H.); 18785237443@163.com (X.D.); 13994341299@163.com (Y.C.); 2 College of Forestry and Horticulture, Hubei Minzu University, Enshi 445000, China; 3 College of Forestry and Landscape Architecture, South China Agricultural University, Guangzhou 510642, China; 13402709191@163.com; 4 College of Biology, Hunan University, Changsha 410082, China; linglei@hnu.edu.cn; 5 State Key Laboratory for Conservation and Utilization of Subtropical Agro-Bioresources, Guangdong Key Laboratory for Innovative Development and Utilization of Forest Plant Germplasm, College of Forestry and Landscape Architecture, South China Agricultural University, Guangzhou 510642, China

**Keywords:** *Phloeosinus cupressi*, climate change, species distribution models, potential distribution, pest management

## Abstract

*Phloeosinus cupressi* Hopkins is an invasive bark beetle that poses a serious threat to *Cupressus* trees, with potential ecological and economic impacts globally. Native to North America, it has spread to Australia and New Zealand, and climate change may further alter its range. Global trade increases the risk of spread, highlighting the need for predictive modeling in management. In this study, we employed CLIMEX and random forest (RF) models to project the potential global distribution of *P. cupressi*, incorporating host distribution data for *Cupressus.* Climatic suitability is concentrated in temperate, subtropical, and Mediterranean zones, including Europe, the U.S., South America, China, Australia, and New Zealand, totaling 10,165.22 × 10^4^ km^2^. Coldest-quarter precipitation (bio19) and annual temperature range (bio7) were identified as the most influential variables. Under RCP6.0 scenarios, suitable areas are projected to expand northward, increasing by ~18%. Regional shifts include contraction in southern Europe and South China, expansion in southern Argentina, southeastern Australia, and coastal New Zealand. Temperature sensitivity is expected to exceed precipitation, enhancing colonization. Due to global *Cupressus* trade, quarantine and monitoring should focus on high-risk regions. Our findings support early detection, long-term monitoring, and control measures for managing *P. cupressi* under climate change.

## Simple Summary

*Phloeosinus cupressi* Hopkins poses a serious threat to forest ecosystems and urban greenery worldwide. While currently absent from China, future climate change could alter its distribution and exacerbate its damage. In this study, we employed CLIMEX and random forest models to predict the global potential distribution of this pest based on its host *Cupressus*, under both current and future climate scenarios, thus quantifying the impacts of climate change. Under future climate projections, the total suitable area is expected to expand. Its native habitat is projected to experience significant northward expansion, posing a major challenge for forest management. Our predictions provide crucial evidence for the current and future global potential distribution of *P. cupressi*, serving as an important reference for identifying regions susceptible to potential infestation by this pest.

## 1. Introduction

*Phloeosinus cupressi* Hopkins is a destructive pest native to North America and mainly distributed in Canada, the United States, Panama, New Zealand, and Australia. In these countries, *P. cupressi* is considered a pest [1,2]. It preferentially reproduces under the bark of dead or dying trees, although it is more commonly found beneath the bark of felled trees or deadwood. Under the current climate conditions, the frequency and severity of this pest infestation are gradually increasing. Adults feed on phloem tissues. Upon emergence, they also bore into small twigs, creating tunnels that extend downward from the center and preserving a thin outer bark layer. Subsequently, numerous bored twigs break off and attract more adults [3,4]. *Cupressus*, native to the temperate regions of the Northern Hemisphere, is the primary target of *P. cupressi* attacks [4]. In New Zealand, the pest poses a threat to *Cupressus macrocarpa*, *Cupressus ariznica*, *Cupressus tortulosa*, *Cupressus arizonica*, and *Chamaetyparis lawsoniana*; In the United States, this beetle harms *Cupressus* spp., *Platycladus orientalis*, *Thuja plicata*, *Thuja occidentalis*, Sequoia sempervirens, and *C. lawsoniana*; In Australia, this pest damages *C. tortulosa* and *Callilres* spp. The pest poses a threat to thousands of hectares of forest in the aforementioned countries [5,6,7,8,9]. Currently, *Cupressus* is widely cultivated in the warm temperate zones of the Northern Hemisphere, Mediterranean coastal regions, Asia (eastern or Eurasia), and North and Central America (western or Americas) [5,6,7,8,9]. *Cupressus* is increasingly selected by countries globally due to its considerable ecological and economic value [8,10,11,12,13,14]. Simultaneously, the ongoing increase in international timber trade likely exacerbates the risk of pest dissemination by *P. cupressi* [15]. Despite the absence of recorded occurrences of *P. cupressi* in certain countries or regions, its potential future distributions should be prioritized.

The global climate is inevitably warming according to the Intergovernmental Panel on Climate Change (IPCC) Sixth Assessment Report (AR6). By the end of the 21st century (2081–2100), global temperatures are projected to increase by 0.2–1.0 °C relative to pre-industrial levels (1850–1900) under a low-emission scenario and by 2.4–4.8 °C under a high-emission scenario [16]. A cross-taxonomic study of Central European insects identified temperature as a strongly correlated predictor for understanding terrestrial species trends [17]. Numerous studies have indicated that the distributions of various terrestrial species, including insects, are shifting toward higher latitudes and elevations due to climate change. Ramalho et al. indicated that the ranges of many species continue to shift toward polar regions or higher elevations, with greater latitudinal shifts observed at higher latitudes. Migration responses to ongoing climate change are stronger in ectotherms than those in endotherms [18]. Chen et al. demonstrated that numerous terrestrial species are shifting their ranges latitudinally or altitudinally in response to climate change, with migration distances being most pronounced in studies characterized by the highest levels of warming [19]. Rubenstein et al. noted that species generally migrate toward higher latitudes and elevations, with temperature serving as the primary climatic driver, while precipitation influences are more complex [20,21,22]. As a highly adaptable pest, the future spread of *P. cupressi* is critical for effective pest management. Therefore, the potential distribution of this pest should be investigated under future climate scenarios.

Species distribution models (SDMs) represent quantitative frameworks that integrate species occurrence data with environmental variables and are typically used to explain and project the potential distributions of species across spatial and temporal dimensions [23]. SDMs are commonly classified into two categories: correlative and mechanistic models. Correlative models, including BIOCLIM, MaxEnt, GARP, and the random forest (RF) algorithm, offer advantages, such as straightforward construction, low computational cost, and strong predictive performance within the environmental parameters defined by the training data [23,24]. They also do not require detailed knowledge of physiology and only require adequate occurrence data; therefore, they have been widely applied. However, the lack of explanatory power regarding mechanisms, reliance on equilibrium assumptions, and high sensitivity to input data quality typically result in low predictive reliability in the context of climate change or novel environmental conditions. Observation biases and sampling irregularities can severely compromise predictive accuracy. Conversely, mechanistic models provide strong explanatory power and excellent extrapolation capabilities. These models can predict outcomes under novel environmental conditions without depending on equilibrium assumptions. The mechanistic hypotheses posited in these models can be validated independently through empirical data or experimental methods. However, these models require numerous parameters and substantial amounts of difficult-to-acquire physiological and ecological data. Therefore, an increasing number of studies have combined correlation and mechanistic models to enhance prediction accuracy and credibility [25]. Currently, the combination of a mechanistic model (i.e., CLIMEX) and correlation models (i.e., RFs) has demonstrated enhanced reliability and ecological plausibility in predictions [26,27,28,29]. Consequently, RFs and CLIMEX were selected to develop models examining the future distributions of *P. cupressi* in this study.

Research on *P. cupressi* has primarily focused on its biology, damage characteristics, and control measures. Monitoring and control strategies for *P. cupressi* include conducting regular surveys of bark beetle infestations and branch damage, collecting subcortical adult specimens, removing infested plant material, and employing the parasitic wasp *Rhaphitelus maculatus* Walker for biological control. These methods offer the advantages of low cost and simple implementation. Biological control serves as an effective component of integrated management to reduce reliance on chemical pesticides. However, due to the impact of climate change, the effectiveness of its prevention and control has been slow to materialize and relatively poor [4]. Thus, one of the critical questions is: whether the *P. cupressi* population can change in response to this epic climate change. To answer this question, we use species distribution models (SDMs) in this study to predict climate change-driven habitat shifts, which may be the key to understanding the prospects of this population. A combination of CLIMEX and RF models was used to predict the potential global distribution of *P. cupressi* under future climate change scenarios. This research had three objectives: (1) to map the potential global distribution of *P. cupressi* under current climatic conditions and identify the key climatic factors influencing its distribution; (2) to project its potential distribution under future climate scenarios and assess possible trends of range expansion or contraction due to global warming; and (3) to establish a theoretical basis for monitoring, early warning, and effective prevention and control measures for the species, thereby offering an important reference for countries in controlling the spread of *P. cupressi*. The findings of this study are anticipated to advance our understanding of the climatic and ecological drivers shaping the global distribution of *P. cupressi* and provide a theoretical basis for developing targeted monitoring, quarantine, and management strategies under future climate change scenarios.

## 2. Materials and Methods

### 2.1. Research Model Framework and Software

#### 2.1.1. CLIMEX Model

The CLIMEX model (version 4.0.2, Hearne Scientific Software, Melbourne, Australia) was used to predict the potential global distribution of *Cupressus* under historical and future climate scenarios. CLIMEX is a dynamic ecological model that simulates the interactions among species growth, survival, geographic distribution, and climatic variables. This approach assesses environmental suitability by integrating climate data with the physiological attributes of the species. The model outputs an Ecoclimatic Index (EI), which indicates the potential growth of a species over time while accounting for environmental stress factors. This index reflects the environmental suitability of a species based on temperature, humidity, and other climatic factors. The EI is derived by combining the annual Growth Index (${\mathrm{GI}}_{A}$) and Stress Index (SI), both of which incorporate temperature and moisture parameters to define habitat suitability [30].

The ${\mathrm{GI}}_{A}$ quantifies the growth potential of a species under favorable climatic conditions and is calculated as the product of the Temperature Index (TI) and Moisture Index (MI). The SI encompasses cold, hot, dry, and wet pressures, providing a cumulative measure of the stress factors that limit survival during unfavorable seasons. Areas with EI values close to zero are deemed unsuitable for the species, whereas those with high EI values are considered highly suitable [31].

In this study, a semi-automated fitting method based on genetic algorithms was used to parameterize the model by adjusting species-specific parameters to fit known *Cupressus* distribution data. The genetic algorithm optimized parameters by maximizing agreement between predicted suitable areas (E.I. > 0) and known occurrence records, while minimizing overprediction beyond the native range. The initial parameter values were derived from existing ecological and physiological data accessed via Google Scholar (https://scholar.google.com). Some parameters referenced the templates provided by CLIMEX with varying geographical representativeness. The fitting process involved fine-tuning these parameters to ensure that the occurrence records matched the predicted habitat suitability [30].

#### 2.1.2. Random Forest (RF) Model

The RF model was implemented with the BioMod2 package in R 4.4.2 and used to model the potential distribution of *P. cupressi* [32,33]. First, a comprehensive dataset of known *P. cupressi* distributions was compiled from published records. A total of 156 pseudo-absent points were randomly generated to minimize sampling bias from occurrence records obtained from diverse sources [34,35]. The model is trained and validated using 10-fold cross-validation. Within each fold, one subset is reserved as validation data to test the model, while the remaining subsets are used for training. For each subset, 70% of the occurrence data is employed to train the model, with the remaining data used to evaluate its predictive performance. Running the model 10 times for cross-validation enhances accuracy, minimizes errors, and delivers more realistic predictions.

The model outputted a raster layer, in which each cell value indicated the probability of *P. cupressi* presence within that cell, with values ranging from 0 to 1. Applying thresholding to this raster layer yielded suitability ratings for *P. cupressi* at each cell location [36]. The output TIF layer was imported into ArcGIS 10.8, and the suitability grades for *P. cupressi* were classified using the natural breakpoint method [37]. This method followed the principles of maximizing between-group variance while minimizing within-group variance to enhance visual separation among suitability classes [38]. It also divided the suitability of the raster layer into five categories: unsuitable, very low, low, medium, and high. Because the threshold for the natural break method could differ based on the actual data distribution across various periods, specific threshold values were not labeled and only suitability categories were displayed on the map.

The RF model used 19 bioclimatic variables sourced from the WorldClim database (https://www.worldclim.org/) as predictors. The Pearson correlation coefficient (r) and the Least Absolute and Signed Selection (LASSO) method were employed to sequentially screen key environmental factors influencing the potential distribution of target species. This dimensionality reduction process also aimed to eliminate model overfitting caused by multicollinearity among environmental factors, thereby enhancing model accuracy. First, Pearson correlation coefficients (r) were calculated between all pairs of the 19 environmental variables. When the correlation coefficient between two variables exceeded 0.8, only one of them was retained. Subsequently, the LASSO algorithm was employed to construct a penalty function, compressing the coefficients of some variables to zero. Finally, all environmental variables with non-zero coefficients were retained as key variables [39]. The model performance was evaluated using several statistical metrics, including the area under the curve (AUC), total sum of squares (TSS), and Kappa coefficient. These metrics were used in combination to evaluate the accuracy of the model predictions. The AUC represents the area under the receiver operating characteristic (ROC) curve and measures the overall classification model performance, with values ranging from 0 to 1 [40]. TSS assesses predictive capability, particularly for imbalanced data, by synthesizing sensitivity and specificity, with values ranging from −1 to 1 [41]. The Kappa value quantifies the difference between model predictions and random guessing, accounting for class imbalance, with values ranging from −1 to 1 [42]. The closer these three values are to 1, the stronger the classification capability of the model and the better its predictive performance.

### 2.2. Data Collection

#### 2.2.1. Historical and Future Climate Data

##### CLIMEX

CLIMEX 4.0 required climate data comprising daily minimum temperature (Tmin), daily maximum temperature (Tmax), monthly precipitation (Rainfall), and relative humidity, all of which were recorded at 9 AM (RH 0900) and 3 PM (RH 1500). These data were collectively converted into CLIMEX .loc and .met file formats. The historical climate variables used were sourced from CliMond’s 10′ resolution gridded data for 1961–1990. The underlying historical climate data were sourced from WorldClim and the CL1.0 and CL2.0 datasets released by the Climate Research Unit [43]. CliMond (http://www.climond.org/) (accessed on 10 January 2025) serves as a comprehensive global climatology database used for bioclimatic modeling purposes. The dataset contains rasterized data for the current and selected future climate scenario layers at 10′ or 30′ spatial resolutions. Data for CLIMEX modeling can be downloaded in standard formats through this platform [34].

The future climate scenario employed in this study was the A1B emission scenario, as outlined in the IPCC AR5. The A1B scenario within the A1 series assumes rapid global economic growth, swift technological advancement, and a balanced energy mix between fossil and non-fossil fuels. In this scenario, greenhouse gas concentrations are projected to increase throughout the 21st century, leading to systematic changes in global mean temperatures and precipitation patterns. Because of its balanced assumptions and widespread adoption, the A1B scenario is widely used in numerous ecological and climate impact simulation studies [44,45,46]. The climate data employed in this study originated from general circulation model (GCM) simulations constructed for the AR5 scenarios and were processed using the CliMond data platform for bioclimatic modeling [47]. The climate data for the 2050s under the A1B scenario were selected to represent the mid-future period from 2041 to 2060.

##### Random Forest (RF)

In this study, the bioclimatic variable layers required to construct the RF prediction model for *P. cupressi* were obtained from the WorldClim Global Climate Database (https://www.worldclim.org/). These layer datasets offered four spatial resolution options: 0.5′, 2.5′, 5′, and 10′. The 10′ resolution bioclimatic layers were selected for RF modeling to ensure consistency with the spatial scale of data used in the CLIMEX model, thereby enabling effective overlay and comparison of model outputs. Current climate data were generated from measurements taken at meteorological stations worldwide. WorldClim employs thin-plate smoothing spline interpolation to process data and execute the interpolation algorithm, yielding high-precision climate layers with global coverage [48].

For future climate projections, the 2050s were chosen as the representative future period. This selection primarily considered the biological characteristics of *P. cupressi*: short life cycle, rapid reproduction rate, and high environmental adaptability. Climate change impacts on its distribution might become evident within a shorter timeframe, making the mid-future time window particularly relevant [49]. In addition, future climate scenarios adopted the representative concentration pathway (RCP) framework established in AR5. This framework comprises four emission scenarios: RCP2.6 (strict mitigation pathway), RCP4.5, RCP6.0 (medium emission pathways), and RCP8.5 (high emission pathway). These emission scenarios correspond to increases in global radiative forcing relative to pre-industrial levels (1750) of 2.6 W/m^2^, 4.5 W/m^2^, 6.0 W/m^2^, and 8.5 W/m^2^ by 2100, respectively [31]. In this study, RCP6.0 was ultimately selected as the future climate projection scheme for the RF model to ensure scenario correspondence and result compatibility with the A1B medium-emission scenario used in CLIMEX modeling.

#### 2.2.2. Known Distributions of *P. cupressi* and *Cupressus*

**Figure 1:**
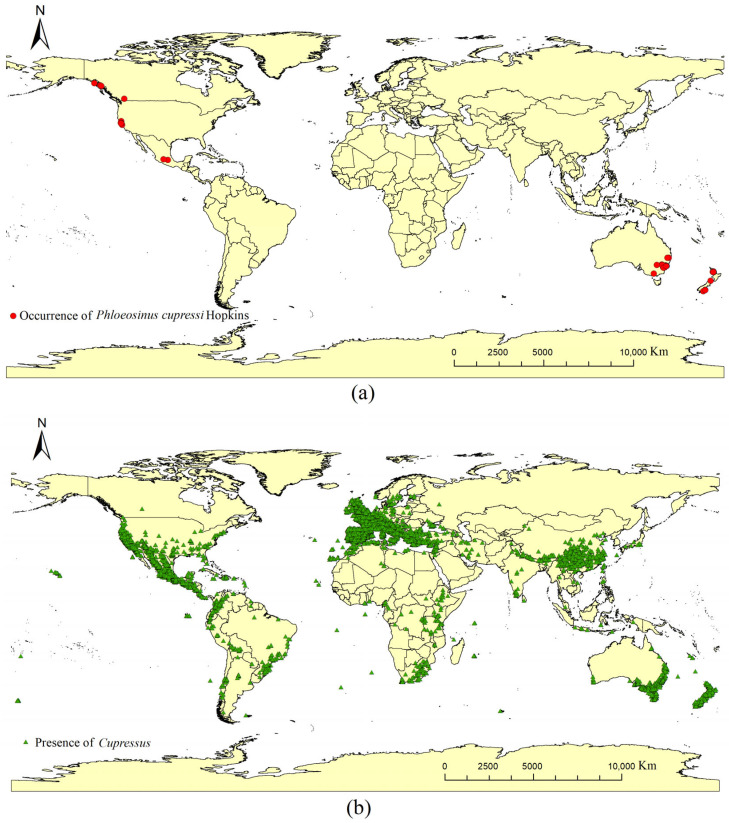
Global presence coordinates of *P. cupressi* (**a**) and *Cupressus* (**b**).

The currently known distributions of *P. cupressi* and *Cupressus* were primarily sourced from the Global Biodiversity Information Facility (GBIF, https://www.gbif.org/). For *P. cupressi*, 204 global distribution coordinates were acquired from GBIF [50]. For host *Cupressus*, 70,109 global distribution coordinates were acquired from GBIF [51]. At present, artificially established cypress stands include both pure stands and mixed forests, with pure stands constituting the majority [52,53]. After eliminating duplicate occurrence records, 52 coordinates for *P. cupressi* were retained (Figure 1a and Table S1). After eliminating duplicate occurrences and filtering the data using the “Spatially Rarefy Occurrence Data” function from the SDM toolbox in ArcGIS 10.8 [54]. Finally, a total of 6625 coordinates for *Cupressus* were retained (Figure 1b).

#### 2.2.3. Parameter Fitting

The parameter setting method was as follows. First, initial parameters were set based on climate types and biological data from native regions (e.g., Western North America, Central America, Northwest Africa, and the Middle East), with reference to templates as needed. Subsequently, 75% of global occurrence records were randomly selected to serve as the training set for parameter adjustment, ensuring that the records fell within the predicted potential distribution area. Finally, the remaining records were used to assess model accuracy and validate prediction precision. This iterative process optimized the model, enhanced the accuracy of global distribution predictions, and effectively simulated environmental and climatic changes [55].

##### Temperature Index

A temperature of −15 °C is regarded as the minimum temperature threshold tolerable by cypress trees. Cypress trees generally survive in northern regions where winter temperatures remain above this threshold. Therefore, DV0 was set to −15 °C. Reference data for the minimum suitable temperature for growth (DV1) and maximum suitable temperature for growth (DV2) were derived from studies of Mediterranean cypress (*Cupressus sempervirens* var. Horizontalis) in two typical distribution zones of Iran. The study revealed the following: Rudbar City had annual average minimum and maximum temperatures of 15 °C and 19.2 °C, respectively; Noshahr City had annual average minimum and maximum temperatures of 12.2 °C and 21.6 °C, respectively [56]. Therefore, the average annual minimum temperature of the two locations (13.6 °C) was used as DV1, whereas their average annual maximum temperature (20.4 °C) was designated as DV2 to model the suitable temperature range for cypress. The native range of *Cupressus* in western North America included the northern Mexico-Arizona border area, characterized by summer temperatures that can reach 45 °C [57]. Therefore, DV3 was established at 45 °C.

##### Moisture Index

*Cupressus* demonstrates considerable drought tolerance. SM0 was set to 0.05, which aligned with the permanent wilting point for deep-rooted plant species [58]. *Cupressus* primarily inhabits temperate, Mediterranean, and semi-arid regions characterized by dry, hot summers and mild winters [57]. The Mediterranean, temperate, and semi-arid templates provided reference settings for SM1 (0.5), SM2 (0.9), and SM3 (1.5), respectively [30].

##### Cold Stress

Mature cypress trees exhibit strong cold tolerance, typically surviving temperatures below −15 °C, with some individuals capable of withstanding temperatures as low as −23 °C [59,60]. However, crown freeze damage is susceptible to extremely low temperatures. Therefore, the cold stress threshold temperature (TTCS) was set to −15 °C, while the cold inhibition accumulation rate (THCS) was established at −0.125 week^−1^ to simulate the critical tolerance of cypress to low temperatures.

##### Heat Stress

Following DV3, the thermal stress temperature threshold (TTHS) was established at 45 °C. Ghodskhah et al. reported that *Cupressus* growth exhibited a significant negative correlation with temperature (r = −0.51) in mountainous and cold-climate regions (e.g., Rudbar), whereas temperature has a lesser impact on growth (r = −0.23) in plains regions (e.g., Noshahr) [56]. The known global distribution of *Cupressus*, as documented in relevant literature records, indicates that its abundance and distribution range in mountainous and cold-climate regions exceed those in plains regions. Therefore, the TTHS was set to 0.015 week^−1^.

##### Dry Stress

Referencing SM0, the minimum soil moisture at which dry stress begins to accumulate (SMDS) was set to 0.05 to reflect the ability of *Cupressus* to maintain minimal physiological activity under extreme drought conditions. Given the notable drought tolerance of the genus *Cupressus*, the drought stress accumulation rate parameter (HDS) was established at −0.005 week^−1^ to more precisely simulate its response to drought stress [58]. This reflects the ecological characteristics of a slower physiological decline and more gradual accumulation of stress effects under drought conditions.

##### Wet Stress

The soil moisture at which wet suppression begins to accumulate (SMWS) was set to 1.5, thereby aligning with SM3. Ghodskhah et al. revealed that in mountainous regions characterized by aridity and strong drainage capacity (e.g., Rudbar), *Cupressus* growth exhibited a significant positive correlation with precipitation (r = 0.94). Conversely, in lowland areas, characterized by elevated soil moisture and reduced drainage capacity (e.g., Noshahr), *Cupressus* growth demonstrated a significant negative correlation with precipitation (r = −0.14) [56]. Based on the known global distribution of *Cupressus* and relevant literature records, its abundance and distribution range in mountainous regions characterized by moderate precipitation and good drainage exceed those in lowlands and extremely humid areas. This indicates that appropriate precipitation facilitates cypress growth; however, it is not adapted to highly humid environments. Therefore, the HWS was set to 0.012 week^−1^.

All fitted parameter values are summarized in Table 1.

### 2.3. Global Potential Distribution of P. cupressi in Relation to Host Cupressus

The RF and CLIMEX prediction results were imported into ArcGIS for further spatial analysis. First, the EI layer was converted into a binary raster; areas with EI = 0 were assigned a value of 0, whereas areas with EI > 0 were assigned a value of 1 (indicating the regions where the host plant *Cupressus* is likely to occur). Subsequently, the RF model prediction results were extracted using the “Extraction by Mask” tool in ArcGIS, employing the areas assigned a value of 1 as a mask layer. This ultimately yielded potentially suitable habitat areas for *P. cupressi* under conditions in which *Cupressus* was present [61].

## 3. Results

### 3.1. Potential Global Distribution of the Host Cupressus Using CLIMEX

**Figure 2:**
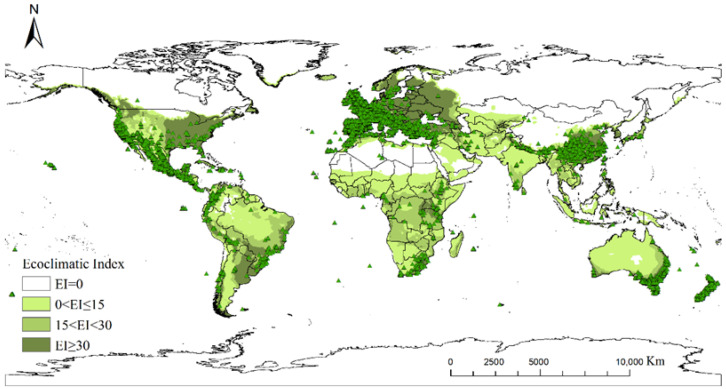
Potential global distribution predicted by CLIMEX (distribution under current climate scenarios) and global presence coordinates of *Cupressus*.

The potential distribution predicted by the CLIMEX model was highly consistent with the global distribution of *Cupressus*, with 99.9% of the observed occurrence points falling within the model-predicted region, indicating that the parameters used possessed good stability and predictive capability (Figure 2). Therefore, these parameters could be used to predict the potential distribution of *Cupressus* with high confidence.

**Figure 3:**
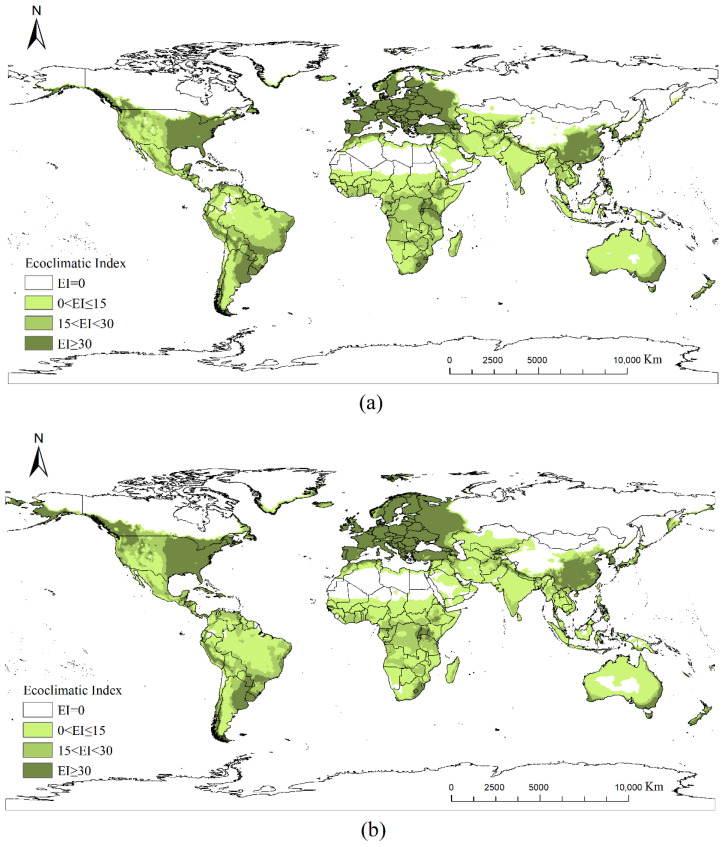
Potential global distribution of *Cupressus* predicted by CLIMEX; (**a**) distribution under current climate scenarios; (**b**) distribution under A1B climate scenarios in 2050.

At present, *Cupressus* occupies a relatively broad range of suitable habitats globally. Regions with an EI ≥ 30 are primarily concentrated in Europe, eastern China, the southeastern United States, southern Brazil, northern Argentina, the southern South Island in New Zealand’s, southeastern Australia, the Korean Peninsula, and southern Japan (Figure 3).

**Figure 4:**
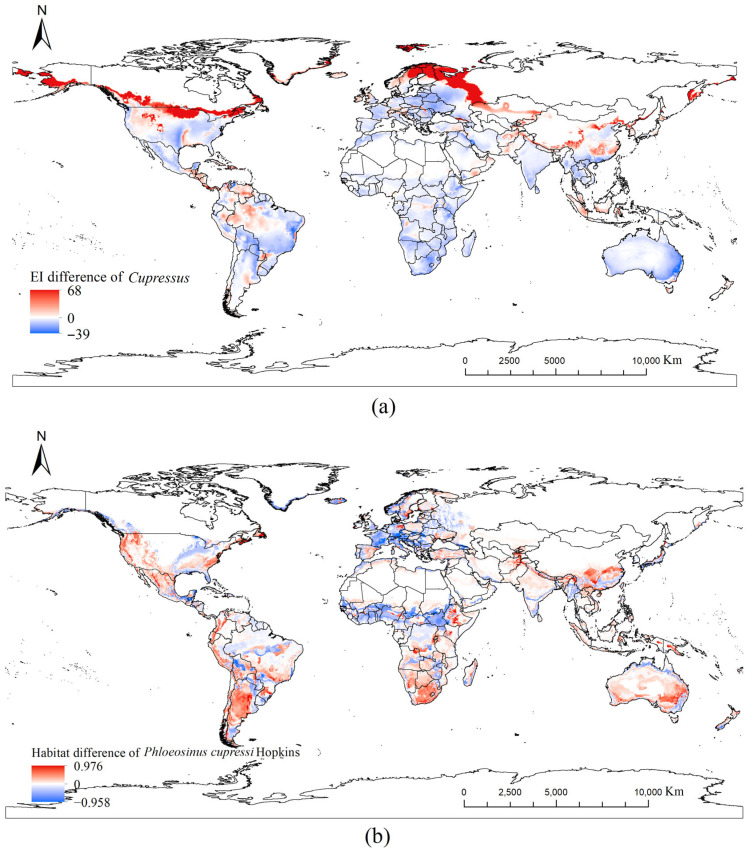
Habitat suitability difference from current to future climate scenarios: (**a**) habitat suitability difference in *Cupressus*; (**b**) habitat suitability difference in *P. cupressi* (the value in each raster is calculated by the future suitability value minus the current suitability value).

Figure 4a illustrates the differences between the current and future potential habitats of *Cupressus* (red and blue areas represent regions with increased and decreased potential habitats, respectively). Under future climatic conditions, *Cupressus* suitable habitat in the Northern Hemisphere shows a clear expansion trend toward higher latitudes, extending into southern Alaska, southern Canada, the northern United States, Northern Europe, western Russia, southern Siberia, northern China, the northern Korean Peninsula, northern Japan, northeastern Turkey, and northern Iran (Figure 4a). In low-latitude tropical and subtropical regions, areas suitable for *Cupressus* growth decreased. Suitable habitats in the Southern Hemisphere exhibited an overall shrinking trend.

### 3.2. Global Potential Distribution of P. cupressi Using Random Forest (RF)

According to the model results under the current climate scenarios, the AUC, Kappa, and TSS evaluation metrics were 0.896, 0.793, and 0.793, respectively. Under future climate scenarios, the metrics increased to 0.933, 0.893, and 0.867, respectively, thereby demonstrating high model accuracy.

**Figure 5:**
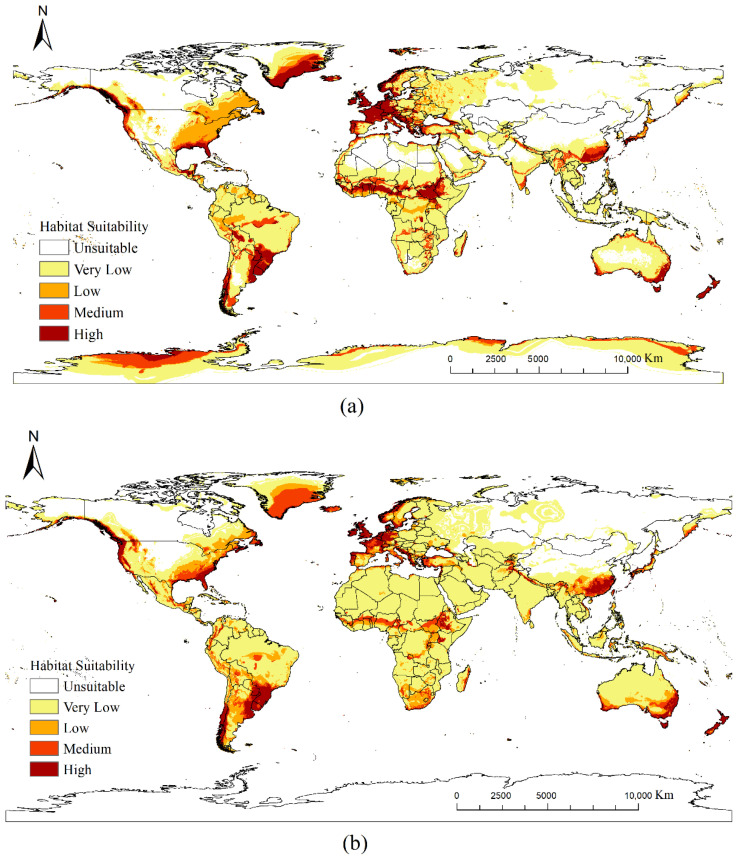
Potential global distribution of *P. cupressi* predicted by Random Forests: (**a**) distribution under current climate scenario; (**b**) distribution under an RCP6.0 climate scenario in 2050.

Globally, regions with high suitability under both current and future climate scenarios were primarily concentrated in the southern and western Andes regions of South America, central and southern France, northeastern Spain, the Mediterranean coast, southwestern Guangxi, southern Yunnan in China, and the southeastern coastal areas of Australia (Figure 5). Medium- or low-suitability areas were predominantly located in subtropical arid or semi-arid transition zones.

**Figure 6:**
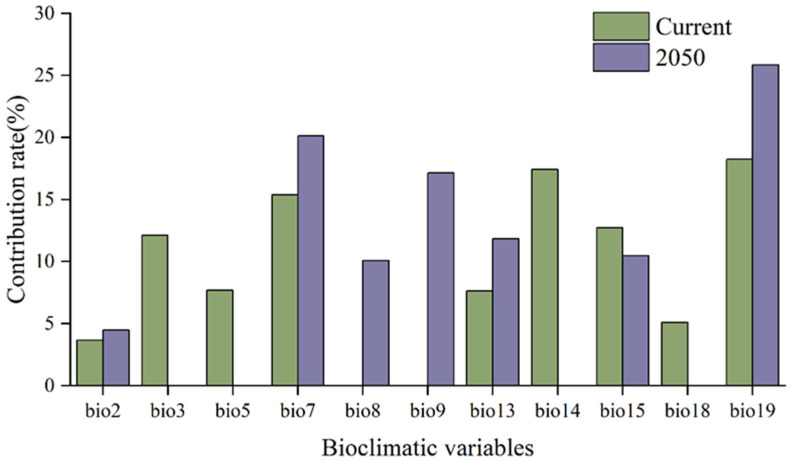
Contribution rates of important variables affecting the potential distribution of *P. cupressi*.

Figure 6 illustrates the contributions of the bioclimatic variables to the model predictions. Under the current climate scenario, the five most significant variables were as follows: coldest quarter precipitation (18.24%), driest month precipitation (17.42%), annual temperature range (15.39%), precipitation seasonality (12.73%), and isothermality (12.11%). Under the RCP6.0 climate scenario, the top five contributing variables were as follows: precipitation during the coldest season (25.86%), annual temperature range (20.12%), average temperature during the driest season (17.13%), precipitation during the wettest month (11.84%), and precipitation seasonality (10.48%). Under the current climate scenario, temperature-related variables collectively contributed 38.86%, whereas precipitation-related variables accounted for 61.13%. Under the RCP6.0 climate scenario, temperature-related variables collectively contributed 51.82%, whereas precipitation-related variables accounted for 48.18%. In summary, *P. cupressi* currently exhibits more sensitivity to precipitation, while its temperature sensitivity is projected to increase in the future, eventually playing a dominant role.

### 3.3. Global Potential Distribution of P. cupressi Overlapping That of the Host Cupressus

**Figure 7:**
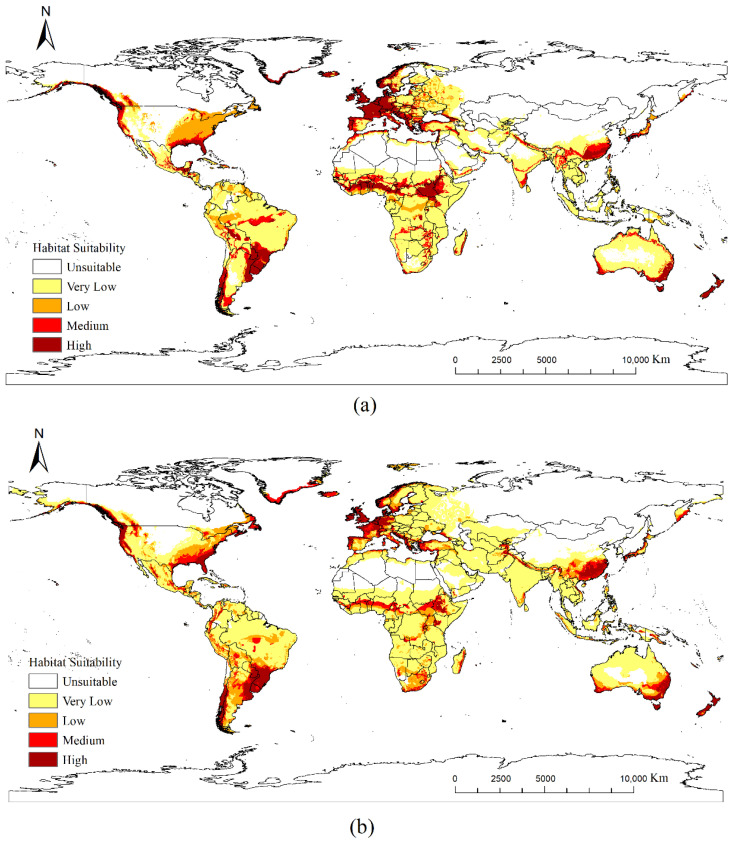
Potential global distribution of *P. cupressi* concerning the host *Cupressus*: (**a**) distribution under current climate scenarios; (**b**) distribution under future climate scenarios in 2050.

Figure 7 shows the global distribution of *P. cupressi* following masking with suitable *Cupressus* regions as hosts. The extraction results demonstrated that nearly all medium- to high-suitability areas were retained.

Figure 4b illustrates the discrepancy between the calculated suitability areas for *P. cupressi* and the future climatic conditions based on Figure 7. Red and blue indicate increased and decreased suitabilities, respectively. Overall, the potential distribution area showed an expanding global trend. The changes in the potential distribution and area differed across continents, as detailed below.

#### 3.3.1. South America

South America exhibited a marked expansion in suitable areas, with an overall improvement in suitability. The suitable land area increases from the current 590.59 × 10^4^ km^2^ to 748.19 × 10^4^ km^2^ (an increase of 26.7%). Brazil’s high-suitability zones will expand significantly, extending from the southeast (São Paulo, Rio) to the southern and inland regions. Western Amazonia will transition from low suitability or unsuitable to medium suitability. Argentina, Uruguay, and Paraguay will generally exhibit shifts from medium- to low-suitability zones to high-suitability zones.

#### 3.3.2. Asia

Asia’s suitable areas are projected to expand markedly northward, with new suitable zones emerging in previously unsuitable high-altitude and high-latitude regions. Conversely, some tropical areas are expected to experience declining suitability. The increase in suitable area ranks second only to South America, expanding from the current 427.26 × 10^4^ km^2^ to 531.14 × 10^4^ km^2^ (a 24.3% increase). Habitability will markedly improve in Central and Northeast China, with suitable areas advancing toward North and Northeast China. In northern India’s Ganges Basin, medium-suitability zones will decrease, whereas high-suitability zones will increase. Some high-suitability areas in Southeast Asia will shift to medium-suitability areas.

#### 3.3.3. Oceania

Suitable areas in Oceania will generally expand, particularly along the southeastern coast and into highland regions. Although the baseline suitable area is small, it will experience the largest increase of 39.4%. Southeastern Australia will experience a substantial improvement in suitability, transforming from predominantly medium- and low-suitability areas to large expanses of high-suitability zones. Low-suitability areas along the eastern coastline will decrease, whereas medium- and high-suitability areas will increase, thereby forming a continuous suitability belt along the coastline. New Zealand’s North Island will experience an increase in the coverage of low- and medium-suitability areas, whereas unsuitable or extremely low-suitability areas will decrease.

#### 3.3.4. North America

The potential habitat area for *P. cupressi* in North America will increase slightly. Under the current scenario, the suitable area is 725.40 × 10^4^ km^2^, whereas under the future scenario, it will be 770.38 × 10^4^ km^2^ (a 6.2% increase). The suitability will continue to improve along the eastern, central, and western coasts of the United States. Medium- to high-suitability areas in Canada are expected to increase significantly. Large expanses of high-suitability areas will emerge in the Central–Southern Pacific coastal region. Low-suitability areas along the Gulf of Mexico coast will decrease, whereas medium-suitability areas will increase.

#### 3.3.5. Europe

Europe’s suitable area will generally decrease, shrinking from 646.95 × 10^4^ km^2^ to 558.83 × 10^4^ km^2^ (a decrease of 13.6%). However, suitability will increase in some regions: Northern England, Northern Germany, and Southern Sweden will shift to suitable areas. Some existing medium-suitability zones (France, Germany, and Poland) will see their suitability ratings increase and connect to contiguous areas.

#### 3.3.6. Africa

Africa’s suitable zones are projected to show a declining trend, with overall shifts toward the highlands, marginal areas, and mid-latitude regions. The suitable area decreases from 650.85 × 10^4^ km^2^ to 577.58 × 10^4^ km^2^ (a decrease of 11.3%). However, suitability increases in some localized areas. West Africa will transition from its current medium-suitability zone to a high-suitability zone, with habitable areas expanding northward. Central Africa is expected to experience a significant increase in suitability levels, with habitable areas expanding southward and eastward. Low- and medium-suitability zones will contract, while new high-suitability zones will emerge in the eastern Congo. Currently, the East African Plateau and southern highlands exhibit almost no habitable areas; however, new habitable zones are expected to emerge in the future.

**Figure 8:**
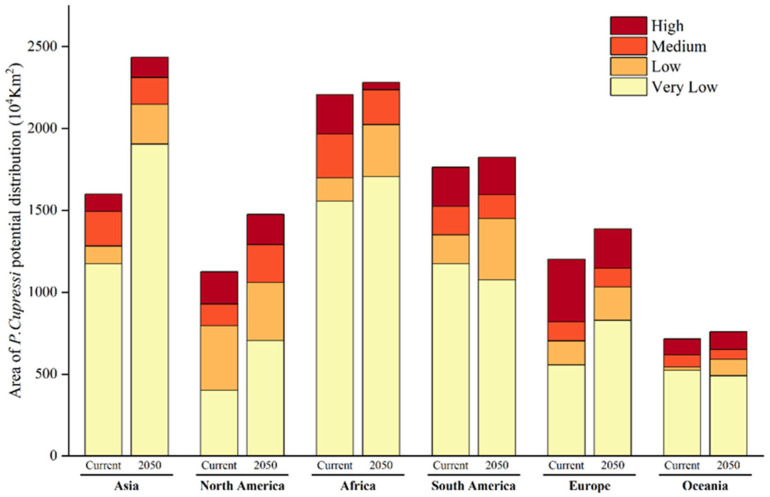
The area of *P. cupressi* habitats of each suitability level in each continent under current climate scenarios and under the RCP6.0 climate scenario in 2050.

Figure 8 shows the current and future areas for each suitability level of *P. cupressi* across continents, calculated based on Figure 6. Within each suitability level, extremely low suitability occupies most areas. Higher suitability levels correspond to smaller areas. Overall, the total current and future suitable areas globally are 8616 × 10^4^ km^2^ and 10,165 × 10^4^ km^2^, respectively. The total suitable area is projected to increase by approximately 17.98% in the future.

## 4. Discussion

### 4.1. Projected Global and Regional Shifts in Climatic Suitability for P. cupressi Under Future Climate Scenarios

The climatic suitability range for *P. cupressi* is predominantly located in Western Europe, Central Europe, the eastern United States, central Mexico, central and southern Argentina, the Mediterranean coast, the Yangtze River Basin and southern regions of China, the southeastern coastal areas of Australia, and the eastern South Island in New Zealand. These regions fall within temperate, subtropical humid, and Mediterranean climate zones. Under future climate scenarios, substantial changes are expected in the following areas: High suitability areas in southern Europe are projected to decrease significantly; High-suitability regions in Southern Argentina are anticipated to exhibit a southward expansion trend. High suitability areas along the southeastern coast of Australia are anticipated to shift southward and become more concentrated within a reduced geographic area. High-suitability areas in the South Island and southern North Island in New Zealand are expected to expand and concentrate in coastal regions. In China, shifts in suitable areas provide critical insights into the survival and spread of this pest species. In the future, the high-suitability areas in the middle and lower reaches of the Yangtze River and parts of South China are expected to experience a substantial reduction, with numerous regions being downgraded to low- or medium-suitability zones. In North China (specifically in Henan, western Shandong, and southern Hebei), low-suitability areas are anticipated to decrease, whereas medium-suitability areas are set to expand. In Southwest China, high-suitability areas in the eastern Yunnan-Guizhou Plateau and Sichuan Basin are expected to decline, with some regions shifting to medium- or low-suitability zones. Southern Northeast China will establish a limited number of low-suitability zones, while adjacent regions in Russia’s Primorsky Krai and northern North Korea will experience similar shifts in suitable areas. Southeast Asian and South Asian countries bordering China, including Vietnam, Laos, Myanmar, India, Bhutan, and Nepal, are set to exhibit declining suitability trends. Notably, the high-suitability zones in northern Vietnam, northern Laos, and northeastern Myanmar will significantly diminish. Northeastern India and Bhutan will shift from high- to medium- and low-suitability zones. Medium-suitability zones in Nepal will decrease, with some areas transitioning to low suitability.

Although high-suitability areas in China’s middle and lower Yangtze River reaches and southwest regions are projected to decrease, high-suitability areas in certain pest-affected areas of the middle and lower Yangtze River (e.g., northern Hunan, central Jiangxi, and southern Anhui) and eastern Sichuan Basin are expected to remain stable. Integrating the current timber trade port locations in China enables the prediction of potential invasion pathways and dispersal directions of *P. cupressi* within the country. Once populations are established at entry ports, *P. cupressi* may follow four transmission routes. First, it may land in southern Liaoning and southern Jilin, spread northward to the northern North China Plain and southward to southeastern Northeast China. This can facilitate transmission or dispersal through land routes to Russia’s Primorsky Krai or northern Korea. Second, it may enter via ports in the Yangtze River Delta and travel along the Yangtze River system to southern Anhui, central Jiangxi, and northern Hunan. These areas, characterized by stable future high-risk provincial distributions, can easily become long-term colonization centers. Third, it may enter via ports in the Guangdong-Hong Kong-Macao Greater Bay Area or Zhanjiang, initially establishing populations in northern Guangdong and northeastern Guangxi before spreading to the middle reaches of the Yangtze River. Simultaneously, it can cross the southwestern border into northern Vietnam and northern Laos. Fourth, it may enter via ports in Myanmar, Laos, and northern Vietnam, initially arriving in southwestern Yunnan before spreading to northeastern Yunnan, Guizhou, and the Sichuan Basin. In summary, blocking entry points, monitoring high-risk landing zones, and implementing rapid containment and quarantine measures are essential for preventing the further spread and invasion of *P. cupressi*.

### 4.2. Shift in Key Climatic Drivers of P. cupressi Distribution

The results indicated that precipitation variables under the current climatic conditions dominated the potential distribution of *P. cupressi*, contributing 61.13% of the total. Under future climate scenarios, temperature-related variables accounted for 51.82% of the potential distribution of *P. cupressi*, reflecting an increase compared with current conditions. Among all the climate variables, coldest quarter precipitation (bio19) and annual temperature range (bio7) exhibited the most substantial changes. Rainfall disrupts normal insect development and reproduction mainly by affecting air humidity, soil moisture, and host plant growth [62]. With the rise in global temperatures, climate change may alter the climate-limiting factors for *P. cupressi*. The interplay of temperature and climate factors affects the distribution and colonization of *P. cupressi*. This species is anticipated to extend its range beyond current precipitation-limited areas into regions with more favorable thermal conditions. Its spatial distribution pattern will increasingly depend on temperature trends rather than solely on precipitation variations [63,64,65,66]. Future global warming may enhance *P. cupressi* habitat suitability, thereby increasing its survival and risk of spreading in high-temperature regions. The impact of bio19 is primarily concentrated in high-latitude regions, including North America and Europe. Under the current scenario, most of these regions demonstrate low to medium suitability. By the 2050s, as climate change progresses, the influence of bio19 substantially intensifies. Some areas in Northern Europe and North America, previously characterized by low suitability, will now exhibit increased suitability and shift from medium to high suitability. The impact of bio7 is greater on tropical regions, such as Africa, South America, and Central America. Under current climatic conditions, most of these areas demonstrate medium to high suitability. However, under future climate scenarios, suitable areas in many tropical and low-latitude regions (e.g., parts of Central America, Africa, and Southeast Asia) are set to expand significantly, with many areas transitioning from medium to high suitability. This expansion is intrinsically linked to the substantial increase in the contribution rate of bio7. In summary, bio19 will predominantly influence the suitability of *P. cupressi* in high-latitude regions, with areas currently deemed of low suitability transitioning to areas of medium or high suitability in the future. In addition, bio7 will significantly impact the suitability of *P. cupressi* in tropical and low-latitude regions, where its current suitability is already high and projected to increase further in the future.

In addition to climate, host plants represent critical factors. Under current climatic conditions, *Cupressus* exhibits high suitability in tropical and subtropical regions, particularly in Africa, South America, Southeast Asia, and parts of Oceania, whereas it shows low suitability in temperate zones, especially in North America, Europe, and parts of Asia. Moreover, *P. cupressi* demonstrates a distribution pattern similar to that of *Cupressus*, with higher suitability in tropical and subtropical regions and lower suitability in temperate zones. Under future climate scenarios, *Cupressus* will exhibit increased suitability in high-latitude regions, particularly in Canada, Russia, and parts of North America, thereby reflecting a northward expansion trend. Previously colder areas will become more suitable for *Cupressus* growth. Conversely, its suitability is anticipated to decline in tropical regions, especially in certain parts of Africa and Asia. The suitability of *P. cupressi* will significantly increase in northern high-latitude regions (e.g., Europe, Canada, and North America) and slightly decrease in tropical regions, aligning with changes in the suitability of *Cupressus* Therefore, changes in the distribution of the host *Cupressus* are expected to directly influence the distribution of *P. cupressi*. The increased suitability for *Cupressus* in high-latitude regions is anticipated to facilitate the expansion of *P. cupressi* distribution into these areas, whereas decreased suitability in tropical regions is set to lead to reduced pest populations in these areas. In addition, human activities may affect the potential distribution of *P. cupressi* [67]. Owing to its long history and high economic value, cypress is extensively cultivated globally. Its presence may extend to regions with previously unsuitable climates, potentially enabling *P. cupressi* emergence in these areas in the future [62]. Furthermore, some cypress populations may remain unrecorded because of artificial planting, potentially influencing predictions regarding the potential distribution of this pest [68]. However, given the complexity of anthropogenic factors, they were excluded from this study and will be analyzed more thoroughly in future research.

### 4.3. Limitations of the Model and Future Management Strategies for P. cupressi

However, the predictive model had certain limitations. In the context of CLIMEX, the biological parameters of species exhibit regional variation; therefore, applying uniform parameters for estimating global potential distributions may compromise model accuracy [69]. In different regions, the physiological parameters of species change with climate variation [70]. Therefore, the spatiotemporal dynamics of these parameters are essential for accurately describing species responses to distribution areas and climate change, thereby improving projection accuracy. In the context of RF, this method demonstrated reliability as a forecasting approach across numerous application domains; however, its fully non-parametric nature limited the explicit integration of physical or biological principles. Consequently, it could not be extrapolated and was limited in its ability to predict long-term trends. Another limitation was the observed prediction errors in certain regions due to the presence of artificially planted cypress trees, as previously noted. Consequently, these aspects should be continuously improved and refined in future research.

Our projections are broadly consistent with previous evidence that international trade and repeated border interceptions are strongly associated with the establishment risk of scolytine beetles, highlighting the importance of major ports and transport corridors for surveillance and prevention [1]. Moreover, the predicted increase in suitability at higher latitudes under mid-century warming agrees with well-documented climate-driven range expansions and enhanced outbreak potential in other bark beetles, where temperature strongly regulates development rate and voltinism [71]. Compared with congeneric *Phloeosinus* pests (e.g., *Phloeosinus aubei* Perris), which have been reported to spread and become damaging in temperate regions and for which lure-based monitoring has been evaluated, our results similarly support prioritizing targeted monitoring in emerging suitable areas and along high-risk pathways [72]. Regions projected to experience significant increases in suitability under future climate scenarios are primarily concentrated in southern Europe (e.g., Portugal, Spain, southern France, and Italy), North Africa, parts of sub-Saharan Africa, selected South American countries (Brazil, Argentina, and Chile), and certain areas of China (East, South, and Southwest China). These regions should be prioritized for pest control efforts. In the native range of *P. cupressi*, biological control measures utilizing local natural enemies or parasites against *P. cupressi* populations can be implemented. In addition, regular monitoring of this bark beetle is essential, and a timely detection plan should be implemented without delay. In non-native areas, enhanced monitoring is essential for the early detection of *P. cupressi* invasions and for implementing rapid control measures to prevent its spread. Rapid interventions using physical, chemical, or biological methods are required for areas that are already infested. Furthermore, strengthening quarantine protocols and strictly monitoring trade and transportation pathways can reduce *P. cupressi* spread via human activities. Improving the health of plants and forests in non-native areas is a crucial strategy. Public education regarding invasive species and their management is necessary to raise awareness and promote prevention.

## 5. Conclusions

This study utilized CLIMEX and RF to predict the global distributions of *P. cupressi* overlapping *Cupressus* under current and future climate scenarios. Results indicate that under current climate conditions, southern and western Europe, central and eastern Africa, southern China, the eastern coastal regions of North America, southeastern South America, eastern Australia, and New Zealand constitute high-suitability regions for *P. cupressi*, covering a total area of approximately 1267.45 × 10^4^ km^2^; these findings are expected to advance our understanding of the climatic and ecological drivers shaping its global distribution and to provide a theoretical basis for developing targeted monitoring, quarantine, and management strategies under future climate change scenarios. Bio19 and bio7 are the key environmental factors that limit the potential distribution of *P. cupressi*. Under future climate scenarios, most highly suitable areas will remain unchanged, while suitability in many regions will undergo varying degrees of change. The total suitable area is projected to increase by approximately 17.98%. Meanwhile, the sensitivity of *P. cupressi* to temperature under future climate scenarios is projected to increase and surpass that of precipitation, greatly increasing the spread and colonization of this pest. The spatial outputs of this study provide actionable support for *P. cupressi* biosecurity. Predicted high-suitability hotspots can be used to prioritize quarantine zoning and to target surveillance at entry points and along pathways associated with *Cupressus* planting materials, wood products, and transport corridors. Areas projected to become suitable under future climates can be treated as early-warning frontiers for proactive monitoring and rapid-response preparedness. Consequently, this study provides guidance for the quarantine, prevention, and control of *P. cupressi*.

## Figures and Tables

**Table 1:** CLIMEX physiological parameter values for *Cupressus*.

CLIMEX Parameter	Final Parameter Value
Temperature requirements	
Lower temperature threshold (°C) (DV0)	−15
Lower optimum temperature (°C) (DV1)	13.6
Upper optimum temperature (°C) (DV2)	20.4
Upper temperature threshold (°C) (DV3)	45
Soil moisture	
Lower soil moisture threshold (SM0)	0.05
Lower optimal soil moisture (SM1)	0.5
Upper optimal soil moisture (SM2)	0.9
Upper soil moisture threshold (SM3)	1.5
Cold stress	
Cold stress temperature threshold (°C) (TTCS)	−15
Cold stress temperature rate (week^−1^)	−0.125
Heat stress (THCS)	
Heat stress temperature threshold (°C) (TTHS)	45
Heat stress temperature rate (week^−1^) (THHS)	0.015
Dry stress	
Dry stress threshold (SMDS)	0.05
Dry stress rate (week^−1^) (HDS)	−0.005
Wet stress	
Wet stress threshold (SMWS)	1.5
Wet stress rate (week^−1^) (HWS)	0.012

## Data Availability

The original contributions presented in this study are included in the article/Supplementary Material. Further inquiries can be directed to the corresponding authors.

## Supplementary Materials

The following supporting information can be downloaded at https://www.mdpi.com/article/10.3390/insects17010077/s1: Table S1: Global occurrence coordinates of *P. cupressi*; Table S2: Global presence coordinates of *Cupressus.*

## References

[1] Brockerhoff E.G., Knížek M., Bain J. (2003). Checklist of indigenous and adventive bark and ambrosia beetles (Curculionidae: Scolytinae and Platypodinae) of New Zealand and interceptions of exotic species (1952–2000). N. Z. Entomol..

[2] Bright D.E. (1976). The Bark Beetles of Canada and Alaska: Coleoptera: Scolytidae.

[3] Hopkins A.D. (1903). Original description of *Phloeosinus cupressi*. Proc. Entomol. Soc. Wash..

[4] Zondag R. (2009). Phloeosinus cupressi, the Cypress Bark Beetle. Forest and Timber Insects in New Zealand No. 3.

[5] Román-Jordán E., Esteban L.G., de Palacios P., Fernández F.G. (2016). Wood anatomy of *Cupressus* and its relation to geographical distribution. IAWA J..

[6] Govaerts R., Farjon A. (2014). World Checklist of Cupressaceae.

[7] Dallimore W., Jackson A.B., Harrison S.G. (1966). A Handbook of Coniferae and Ginkgoaceae.

[8] Wolf C.B., Wagener W.E. (1948). The New World Cypresses.

[9] Bailey L.H. (1923). The Cultivated Evergreens: A Handbook of the Coniferous and Most Important Broad-Leaved Evergreens Planted for Ornament in the United States and Canada.

[10] Yan W., Xiang Y., Gao M., Deng R., Sun Y., Wan R., Pan X., Li W., Zhong Y. (2024). Phenotypic diversity and provenance variation of *Cupressus funebris*: A case study in the Sichuan Basin, China. Peerj.

[11] Bergsten U., Sundberg M. (1990). IDS-sedimentation of *Cupressus lusitanica* seeds. Tropical Tree Seed Research.

[12] Von Carlowitz P.G. (1986). Multipurpose Tree and Shrub Seed Directory.

[13] Bolotin M. (1964). Segregation in progenies of *Cupressus sempervirens* L.. La-Yaaran.

[14] Bannister M. (1962). Prospects for selection in the cypresses. N. Z. J. For..

[15] Li K., Liu H., Li H., Tan J., Zhang Y., Yuan S., Wang X., Lin L., Hu X. (2013). Morphological Features, Harmfulness of an Alien Quarantine Pest *Phloeosinus cupressi*. Hubei Agric. Sci..

[16] Intergovernmental Panel on Climate Change (2023). Future Global Climate: Scenario-based Projections and Near-term Information. Climate Change 2021—The Physical Science Basis.

[17] Bowler D.E., Hof C., Haase P., Kröncke I., Schweiger O., Adrian R., Baert L., Bauer H.-G., Blick T., Brooker R.W. (2017). Cross-realm assessment of climate change impacts on species’ abundance trends. Nat. Ecol. Evol..

[18] Ramalho Q., Vale M.M., Manes S., Diniz P., Malecha A., Prevedello J.A. (2023). Evidence of stronger range shift response to ongoing climate change by ectotherms and high-latitude species. Biol. Conserv..

[19] Chen I., Hill J.K., Ohlemüller R., Roy D.B., Thomas C.D. (2011). Rapid range shifts of species associated with high levels of climate warming. Science.

[20] Rubenstein M.A., Weiskopf S.R., Bertrand R., Carter S.L., Comte L., Eaton M.J., Johnson C.G., Lenoir J., Lynch A.J., Miller B.W. (2023). Climate change and the global redistribution of biodiversity: Substantial variation in empirical support for expected range shifts. Environ. Evid..

[21] Duffy K., Gouhier T.C., Ganguly A.R. (2022). Climate-mediated shifts in temperature fluctuations promote extinction risk. Nat. Clim. Chang..

[22] Moir M.L., Hughes L., Vesk P.A., Leng M.C. (2014). Which host-dependent insects are most prone to coextinction under changed climates?. Ecol. Evol..

[23] Elith J., Leathwick J.R. (2009). Species distribution models: Ecological explanation and prediction across space and time. Annu. Rev. Ecol. Evol. Syst..

[24] Kearney M.R., Wintle B.A., Porter W.P. (2010). Correlative and mechanistic models of species distribution provide congruent forecasts under climate change. Conserv. Lett..

[25] Dormann C.F., Schymanski S.J., Cabral J., Chuine I., Graham C., Hartig F., Kearney M., Morin X., Römermann C., Schröder B. (2012). Correlation and process in species distribution models: Bridging a dichotomy. J. Biogeogr..

[26] Early R., Rwomushana I., Chipabika G., Day R. (2022). Comparing, evaluating and combining statistical species distribution models and CLIMEX to forecast the distributions of emerging crop pests. Pest Manag. Sci..

[27] Yoon S., Kim S.H., Lee W.H. (2026). Spatial prediction of *Melanoplus differentialis* using an ensemble of multiple species distribution models. Insect Conserv. Divers..

[28] Shabani F., Kumar L., Ahmadi M. (2016). A comparison of absolute performance of different correlative and mechanistic species distribution models in an independent area. Ecol. Evol..

[29] Hayat U., Shi J., Wu Z., Rizwan M., Haider M.S. (2024). Which SDM Model, CLIMEX vs. MaxEnt, Best Forecasts *Aeolesthes sarta* Distribution at a Global Scale under Climate Change Scenarios?. Insects.

[30] Kriticos D.J., Maywald G.F., Yonow T., Zurcher E.J., Herrmann N.I., Sutherst R. (2015). CLIMEX Version 4: Exploring the Effects of Climate on Plants, Animals and Diseases. CSIRO.

[31] Sutherst R.W., Maywald G. (1985). A computerised system for matching climates in ecology. Agric. Ecosyst. Environ..

[32] R Core Team (2010). R: A Language and Environment for Statistical Computing.

[33] Thuiller W., Georges D., Gueguen M., Engler R., Breiner F., Lafourcade B., Patin R., Blancheteau H. biomod2: Ensemble Platform for Species Distribution Modeling. https://cran.r-project.org/web/packages/biomod2/index.html.

[34] Chen X., Xiao K., Deng R., Wu L., Cui L., Ning H., Ai X., Chen H. (2024). Projecting the future redistribution of *Pinus koraiensis* (Pinaceae: Pinoideae: Pinus) in China using machine learning. Front. For. Glob. Chang..

[35] Elith J., Graham C.H., Anderson R.P., Dudík M., Ferrier S., Guisan A., Hijmans R.J., Huettmann F., Leathwick J.R., Lehmann A. (2006). Novel methods improve prediction of species’ distributions from occurrence data. Ecography.

[36] Georgiades P., Proestos Y., Lelieveld J., Erguler K. (2023). Machine learning modeling of Aedes albopictus habitat suitability in the 21st century. Insects.

[37] Li Y., Wan Y., Lin W., Ernstsons A.S., Gao L. (2021). Estimating potential distribution of sweetgum pest *Acanthotomicus suncei* and potential economic losses in nursery stock and Urban Areas in China. Insects.

[38] Hou C., Xie Y., Zhang Z. (2022). An improved convolutional neural network based indoor localization by using Jenks natural breaks algorithm. China Commun..

[39] Xiao K., Deng R., Chen X., Yu C., Wu L., Ning H., Chen H. (2025). Projection of the Climate-Suitable Area of the Invasive Pest *Phoracantha semipunctata* (Coleoptera: Cerambycidae: Phoracantha) and Its Ability to Continue to Expand in China. Insects.

[40] Fielding A.H., Bell J.F. (1997). A review of methods for the assessment of prediction errors in conservation presence/absence models. Environ. Conserv..

[41] Allouche O., Tsoar A., Kadmon R. (2006). Assessing the accuracy of species distribution models: Prevalence, kappa and the true skill statistic (TSS). J. Appl. Ecol..

[42] McHugh M.L. (2012). Interrater reliability: The kappa statistic. Biochem. Med..

[43] He S., Ge X., Wang T., Wen J., Zong S. (2015). Areas of potential suitability and survival of Dendroctonus valens in china under extreme climate warming scenario. Bull. Entomol. Res..

[44] Record S., Charney N., Zakaria R., Ellison A.M. (2013). Projecting global mangrove species and community distributions under climate change. Ecosphere.

[45] Falloon P., Dankers R., Betts R., Jones C., Booth B., Lambert F. (2012). Role of vegetation change in future climate under the A1B scenario and a climate stabilisation scenario, using the HadCM3C Earth system model. Biogeosciences.

[46] Chen W., Jiang Z., Li L. (2011). Probabilistic projections of climate change over China under the SRES A1B scenario using 28 AOGCMs. J. Clim..

[47] Nakicenovic N., Alcamo J., Davis G., Vries B., Fenhann J., Gaffin S., Gregory K., Grubler A., Jung T.Y., Kram T. (2000). Special Report on Emissions Scenarios.

[48] Fick S.E., Hijmans R.J. (2017). WorldClim 2: New 1-km spatial resolution climate surfaces for global land areas. Int. J. Climatol..

[49] Skendžić S., Zovko M., Živković I.P., Lešić V., Lemić D. (2021). The impact of climate change on agricultural insect pests. Insects.

[50] GBIF Occurrence Download. https://doi.org/10.15468/dl.kyu2xe.

[51] GBIF Occurrence Download. https://doi.org/10.15468/dl.k8dj3j.

[52] Lyu Q., Luo Y., Liu S., Zhang Y., Li X., Hou G., Chen G., Zhao K., Fan C., Li X. (2022). Forest gaps alter the soil bacterial community of weeping cypress plantations by modulating the understory plant diversity. Front. Plant Sci..

[53] Wang Y., Chen S., He W., Ren J., Wen X., Wang Y., Li X., Chen G., Feng M., Fan C. (2021). Shrub diversity and niche characteristics in the initial stage of reconstruction of low-efficiency cupressus funebris stands. Forests.

[54] Brown J.L. (2014). SDM toolbox: A python-based GIS toolkit for landscape genetic, biogeographic and species distribution model analyses. Methods Ecol. Evol..

[55] Ge X., He S., Zhu C., Wang T., Xu Z., Zong S. (2019). Projecting the current and future potential global distribution of *Hyphantria cunea* (Lepidoptera: Arctiidae) using CLIMEX. Pest Manag. Sci..

[56] Ghodskhah Daryaei M., Ahoo Ghalandari N., Torkaman J. (2021). Investigation the effect of climate variables on growth patterns of the cypress (*Cupressus sempervirens* Var. *Horizontalis*) trees in Noshahr and Rudbar habitats. J. Plant Res..

[57] Frezza C., De Vita D., Sciubba F., Toniolo C., Tomassini L., Nicoletti M., Franceschin M., Guiso M., Bianco A., Serafini M. (2022). There is not only *Cupressus sempervirens* L.: A review on the phytochemistry and bioactivities of the other *Cupressus* L. species. Appl. Sci..

[58] Tadros M.J., Alqudah A.M., Arabiat Y.S. (2010). Comparative study between *Cupressus sempervirens* and *Cupressus arizonica* in seed germination and seedling vigour. Crop Res..

[59] Raddi P., Panconesi A. (1989). Genetic variability of tolerance to cold in *Cupressus sempervirens* progenies. Silvae Genet.

[60] Larcher W. (2001). Ökophysiologie der Pflanzen: Leben, Leistung und Streßbewältigung der Pflanzen in ihrer Umwelt.

[61] Yoon S., Lee W.-H. (2023). Assessing potential European areas of Pierce’s disease mediated by insect vectors by using spatial ensemble model. Front. Plant Sci..

[62] Cui J., Yin J., Dong L., Gao Y., Shi S., Zou J., Li W., Wang Y. (2025). Impact of Temperature and Soil Moisture on the Life Cycle of the Strawberry Pest Priophorus fulvostigmatus and Its Control. Insects.

[63] Rosace M., Björklund N., Boberg J., Bradshaw C., Camac J., Damus M., Kompas T., Li C., MacLeod A., Maggini R. (2024). Including climate change in pest risk assessment: Current practices and perspectives for future implementation. EPPO Bull..

[64] Subedi B., Poudel A., Aryal S. (2023). The impact of climate change on insect pest biology and ecology: Implications for pest management strategies, crop production, and food security. J. Agric. Food Res..

[65] Harvey J.A., Heinen R., Gols R., Thakur M.P. (2020). Climate change-mediated temperature extremes and insects: From outbreaks to breakdowns. Glob. Chang. Biol..

[66] Yan Y., Wang Y., Feng C., Wan P., Chang K. (2017). Potential distributional changes of invasive crop pest species associated with global climate change. Appl. Geogr..

[67] Williams I.S., Jones T.H., Hartley S.E. (2001). The role of resources and natural enemies in determining the distribution of an insect herbivore population. Ecol. Entomol..

[68] Amini T., Ejtehadi H., Djamali M., Guibal F. (2020). The world’s easternmost natural stands of *Cupressus sempervirens* L.(Cupressaceae) in the Hyrcanian Forest of Iran. J. Mediterr. Ecol..

[69] Guisan A., Petitpierre B., Broennimann O., Daehler C., Kueffer C. (2014). Unifying niche shift studies: Insights from biological invasions. Trends Ecol. Evol..

[70] Chown S.L., Hoffmann A.A., Kristensen T.N., Angilletta M.J., Stenseth N.C., Pertoldi C. (2010). Adapting to climate change: A perspective from evolutionary physiology. Clim. Res..

[71] Jönsson A.M., Harding S., Krokene P., Lange H., Lindelöw Å., Økland B., Ravn H.P., Schroeder L.M. (2011). Modelling the potential impact of global warming on Ips typographus voltinism and reproductive diapause. Clim. Chang..

[72] Fiala T., Holuša J. (2018). Occurrence of the invasive bark beetle Phloeosinus aubei on common juniper trees in the Czech Republic. Forests.

